# The relationships of vitamin D, vitamin D receptor gene polymorphisms, and vitamin D supplementation with Parkinson’s disease

**DOI:** 10.1186/s40035-020-00213-2

**Published:** 2020-09-01

**Authors:** Lingling Lv, Xuling Tan, Xinke Peng, Rongrong Bai, Qile Xiao, Ting Zou, Jieqiong Tan, Hainan Zhang, Chunyu Wang

**Affiliations:** 1 Department of Neurology, The Second Xiangya Hospital, Central South University, Changsha, 410011 China; 2 Center for Medical Genetics, School of Life Sciences, Central South University, Changsha, 410078 China; 3 Hunan Key Laboratory of Animal Models for Human Diseases, Central South University, Changsha, 410078 China; 4 Hunan Key Laboratory of Medical Genetics, Central South University, Changsha, 410078 China; 5 Department of Medical Genetics, The Second Xiangya Hospital, Central South University, Changsha, 410011 China

**Keywords:** Parkinson’s disease, Vitamin D, VDR gene polymorphisms, Vitamin D_3_ supplementation, Neuroprotective, Dopaminergic neurotransmission

## Abstract

In recent years, many studies have investigated the correlations between Parkinson’s disease (PD) and vitamin D status, but the conclusion remains elusive. The present review focuses on the associations between PD and serum vitamin D levels by reviewing studies on the associations of PD with serum vitamin D levels and vitamin D receptor (VDR) gene polymorphisms from PubMed, Web of Science, Cochrane Library, and Embase databases. We found that PD patients have lower vitamin D levels than healthy controls and that the vitamin D concentrations are negatively correlated with PD risk and severity. Furthermore, higher vitamin D concentrations are linked to better cognitive function and mood in PD patients. Findings on the relationship between VDR gene polymorphisms and the risk of PD are inconsistent, but the *FokI* (C/T) polymorphism is significantly linked with PD. The occurrence of *FokI* (C/T) gene polymorphism may influence the risk, severity, and cognitive ability of PD patients, while also possibly influencing the effect of Vitamin D_3_ supplementation in PD patients. In view of the neuroprotective effects of vitamin D and the close association between vitamin D and dopaminergic neurotransmission, interventional prospective studies on vitamin D supplementation in PD patients should be conducted in the future.

## Introduction

Parkinson’s disease (PD) is the most common type of parkinsonism, a syndrome characterized by bradykinesia, postural instability, rigidity, and resting tremor [1]. The pathophysiological cause of PD is the progressive loss of dopaminergic (DA) neurons in the substantia nigra (SN) of the midbrain [2–4] and the formation of Lewy bodies, which are neuronal inclusions mainly consisting of α-synuclein protein aggregations [5–7]. In high-income countries, the annual incidence of PD is 14 per 100,000 in the general population, and rises to 160 per 100,000 in the population aged 65 years or older [8]. A systematic review estimated that there were 6.1 million PD patients worldwide in the year 2016, a significant increase from 2.5 million in 1990, with further projected increases in the future. Moreover, the increase cannot entirely be explained by the growth of number of older people [9]. The etiology of PD remains unknown and is presumably multifactorial [10]. The exact mechanism of neurodegeneration in PD is not yet fully elucidated, but it likely involves a series of events including interactions between genetic and environmental factors, oxidative stress, mitochondrial dysfunction, inflammation, immune regulation, and others [11–22]. Due to the unclear etiology, no medications have been proven to cure PD [1]. Therefore, there is a critical demand for new and targeted drugs that focus on protecting DA neurons from degeneration in PD.

**Fig. 1:**
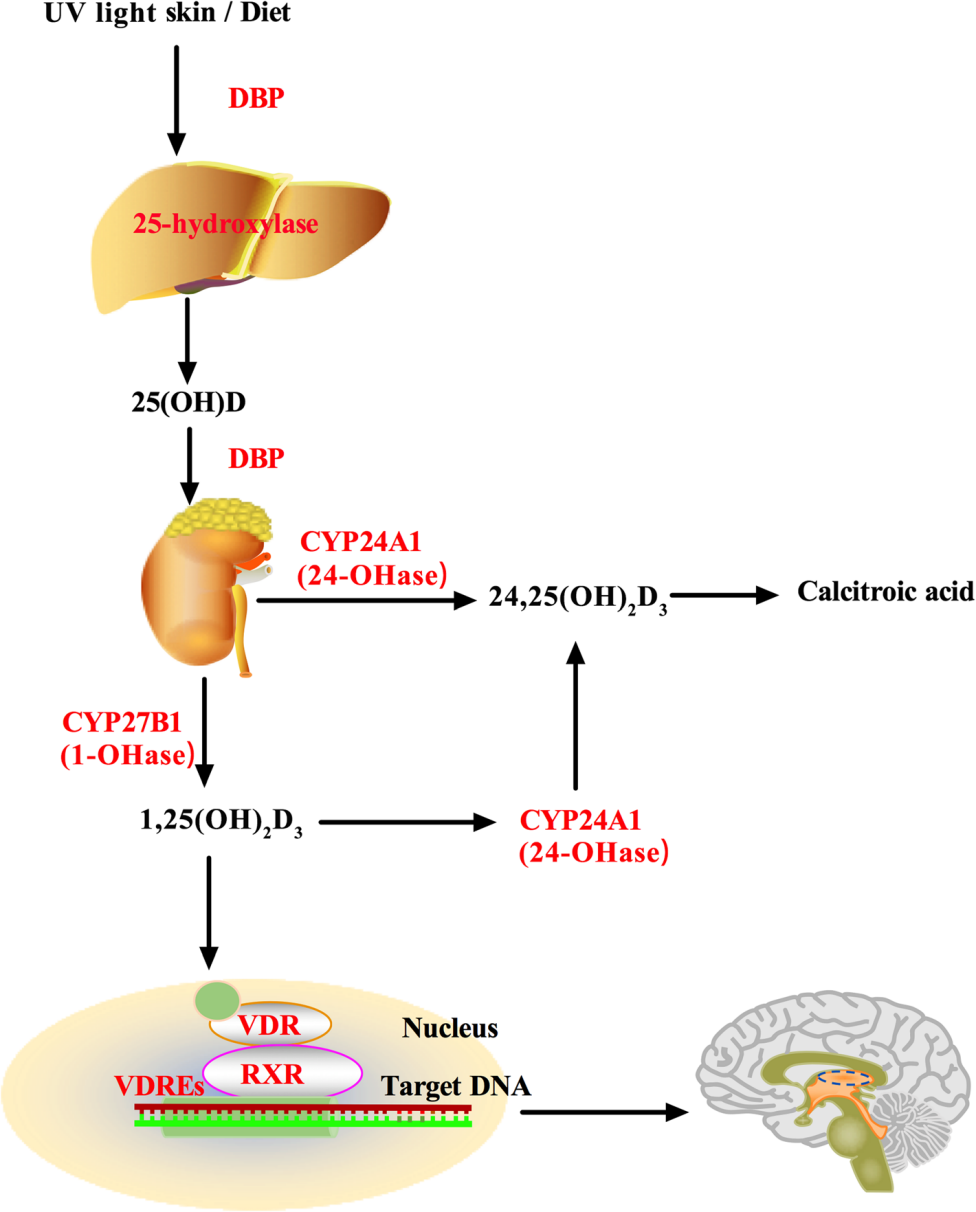
Vitamin D metabolism. DBP, Vitamin D-binding protein; RXR, retinoid X receptor; VDREs, vitamin D response elements; VDR, vitamin D receptor; 1-OHase, 25-hydroxyvitamin D-1α- hydroxylase; 24-OHase, 25-hydroxyvitamin D-24-hydroxylase

Vitamin D obtained via sun exposure or through the diet is converted by 25-hydroxylase mainly located in the liver into 25-hydroxyvitamin D (25(OH)D), the major circulating form of vitamin D. The 25(OH)D can be used as a serum marker to measure vitamin D levels in PD patients, however, it is biologically inactive and must be transformed into the active form 1,25-dihydroxyvitamin D_3_ (1,25(OH)_2_D_3_) by 25-hydroxyvitamin D-1α-hydroxylase (CYP27B1) in the kidney. Increased concentrations of 1,25(OH)_2_D_3_ can raise the expression of 25-hydroxyvitamin D-24-hydroxylase (CYP24A1) to catabolize 1,25(OH)_2_D_3_ into calcitroic acid [23–25]. The biological functions of 1,25(OH)_2_D_3_ are mediated by the vitamin D receptor (VDR), a member of the nuclear receptor superfamily of transcription factors [25, 26]. Upon ligand binding, the VDR interacts with the retinoid X receptor (RXR) to form a heterodimer, which then binds to vitamin D response elements (VDREs) in target genes to promote their expression [27, 28]. It has been predicted that 1,25(OH)_2_D_3_ regulates more than 200 genes, influencing a variety of cellular processes (Fig. 1) [29]. There is ample evidence from in vitro and animal studies that vitamin D plays an important role in cell proliferation and differentiation, neurotrophic regulation and neuroprotection, neurotransmission, immune regulation, and neuroplasticity [30–33]. Studies have confirmed the presence of vitamin D metabolites, their metabolizing enzymes CYP27B1 and CYP24A1, as well as VDR in the human brain. This indicates that the human brain can regulate 1,25(OH)_2_D_3_ levels, and vitamin D may play a key role in the maintenance of normal nervous system function [34, 35]. Moreover, VDR and CYP27B1 expression is most abundant in the substantia nigra (SN; a brain region rich in dopaminergic neurons) according to immunofluorescence [36]. Studies have also found that the earliest time of VDR expression in the midbrain is on embryonic day 12 (E12), which coincides with the time of development of a majority of dopaminergic neurons in the SN region [37, 38]. Vitamin D is a fat-soluble hormone that can pass the blood-brain barrier, which supports its significance in the central nervous system [39].

Considering the neuroprotective effect of vitamin D in the human brain, researchers have proposed a ‘two-hit hypotheses’ to explain how low vitamin D levels make the nervous system more susceptible to secondary harmful effects, which may aggravate the development of diseases such as PD and cerebrovascular disease [40–42]. Notably, many studies have indicated that vitamin D metabolism may be directly or indirectly related to the pathogenesis of PD [30, 31, 33, 39, 42–44]. Further, vitamin D can act as a neuroprotective agent to provide partial protection for DA neurons [45]. Accordingly, inadequate vitamin D status may play a significant role in PD, resulting in a progressive loss of DA neurons in the human brain [46]. However, experimental data from the Asymptomatic Parkinson Associated Vitamin D Intake Risk Syndrome cohort were not consistent with the hypothesis that chronically inadequate levels of vitamin D threaten the integrity of the DA system, leading to the pathogenesis of PD [47]. The conundrum of the connection between serum vitamin D levels and PD therefore remains unsolved. In this paper, we review the serum vitamin D concentrations in PD patients, the relationships of serum vitamin D concentrations and VDR gene polymorphisms with PD risk, the relationship between serum vitamin D concentrations and clinical manifestations of PD patients, as well as the preventive and therapeutic roles of vitamin D in PD.

## Main text

### PD patients often have low serum vitamin D concentrations

Vitamin D insufficiency is prevalent in the elderly worldwide [48], and it is also a common health problem in neurodegenerative diseases such as PD and Alzheimer’s disease (AD). Recently, it has been reported that vitamin D insufficiency is more common among PD patients than healthy controls [49–51]. If the insufficiency of vitamin D is a consequence of neurodegenerative disease, the incidence of vitamin D insufficiency in AD and PD patients should be similar, but a study revealed that vitamin D insufficiency in PD patients was more pronounced than that in AD patients and controls (55% *versus* 41% and 36%, respectively) [52]. The 1,25(OH)_2_D_3_ levels were normal in all PD patients, whereas the serum levels of 25(OH)D were insufficient (< 20 ng/mL) in 49% of patients in a prospective cohort study [53]. This can be explained by the fact that circulating 25(OH)D levels are 1000 times higher than 1,25(OH)_2_D_3_, and that the 25(OH)D can be converted into 1,25(OH)_2_D_3_ by 1a-OHase [29, 53].

PD patients experience mobility problems more frequently, and the typical course of PD is longer than that of AD. Both factors may decrease sunlight exposure, thus reducing the cutaneous synthesis of vitamin D. Many studies have reported that the more severe the motor symptoms, the lower the 25(OH)D concentrations in PD patients [53–57]. The reduced mobility and sunlight deprivation may be responsible for the higher incidence of vitamin D deficiency in PD patients. However, compared with controls, there are significantly lower levels of 25(OH)D in PD patients with sufficient sunlight exposure [51]. This may be because that as some vitamin D should be from the diet [29], the gastrointestinal dysfunction in PD patients may result in chronically inadequate vitamin D intake [58, 59]. Interestingly, a longitudinal cohort study discovered that the 25(OH)D concentrations were slightly increased over the study period, which means that these patients did not have digestive dysfunction. The study also reported that there was a high incidence of vitamin D insufficiency in subjects with early PD who did not require symptomatic therapy [60].

However, another study showed that compared with controls, the PD patients had slightly yet not significantly lower serum vitamin D concentrations [61]. There may be two factors that affect the results. First, the case-control study was conducted in the Faroe Islands at a high latitude and with harsh climate and frequent cloud cover, which may have decreased sunlight exposure. Second, Faroese food is not rich in vitamin D, which resulted in the common vitamin D insufficiency in the Faroe Islands.

### The relationship between vitamin D deficiency and PD risk

Lower vitamin D levels may be a result of PD due to the limited mobility and digestive symptoms of PD patients. However, several studies suggested that vitamin D deficiency may be associated with the etiology of PD [46, 54]. A study showed that the prevalence of vitamin D deficiency was higher among patients with PD, even if they had normal ambulation and gastrointestinal functions [60]. Newmark and colleagues concluded that chronic vitamin D deficiency is likely to be linked to the pathogenesis or progression of PD rather than only being a consequence of the disease [46, 54]. This hypothesis was also supported by a 29-year prospective study in Finland, which confirmed that those who had higher vitamin D concentrations were less likely to develop PD. When comparing probands in the highest and the lowest quartiles of vitamin D levels, the relative risk of PD was 0.33 for the highest quartile (95% confidence interval, 0.14–0.80) [62]. In line with these findings, a large case-control sample study revealed a negative correlation between PD risk and the level of 25(OH)D, and additionally showed an inverse correlation between 25(OH)D_2_ and PD [59]. Moreover, Danish and Chinese case-control studies both suggested that outdoor work can reduce the risk of PD in later life [63–65]. One possible protective mechanism of outdoor work is to increase sunlight exposure, which contributes to vitamin D_3_ synthesis in the skin. Interestingly, a nationwide ecological study in France showed that increasing sunlight exposure can reduce the risk of PD in the young population [66]. However, a population-based prospective study with 17 years of follow-up and a Mendelian randomization study did not explore the association between 25(OH)D concentrations and the prevalence of PD [67, 68].

### The relationship between VDR gene polymorphisms and PD risk

The VDR is the key mediator of the functions of vitamin D. A transcriptome-wide scan indicated that the VDR gene expression is increased in the blood cells of early-stage PD patients [69]. Consequently, it is reasonable that the VDR polymorphic variants might also have an effect on the pathogenesis of PD. In recent years, the polymorphisms of *BsmI* (rs1544410), *FokI* (rs10735810), *ApaI* (rs7975232), and *TaqI* (rs731236) have been most widely studied in research on the correlation between VDR gene variants and PD, but the results were inconsistent [70–72]. A polymerase chain reaction-based restriction analysis of VDR gene polymorphisms in Korea indicated that the *BsmI* (B/b) polymorphism is a candidate allele influencing the pathogenesis of PD. The study further showed that the bb genotype was more common in the group with predominant postural instability and gait disorders than in the tremor-predominant group and the healthy controls [73]. In addition, Hungarian, Japanese and Chinese studies suggested that the *FokI* (C/T) polymorphism located in exon 2 in the 5′ coding region of the gene was significantly linked with PD, and the C allele can increase the risk of PD [74–77].

The most significant start codon polymorphism of the VDR gene is the functional *FokI* polymorphism, which results in different translation initiation sites, one producing a long version of the VDR protein (the T-allele) and the other producing a protein shortened by three amino acids (the C-allele) [70]. In spite of the small difference, the functional characteristics of the two forms of VDR (C-VDR and T-VDR) are significantly different. Compared with T-VDR, the C-VDR has a better capacity for intestinal calcium absorption [70, 78]. Therefore, the C allele may forecast higher vitamin D levels and reduce the risk of PD. However, research findings have suggested that the C allele is a risk factor for PD rather than being a protective factor [74–77]. Suzuki et al. revealed that there was a stronger association of the *FokI* CC genotype with milder forms of PD (odds ratio, 0.32; 95% confidence interval, 0.16–0.66) [53]. Moreover, the Parkinson Environment Gene study, a population-based case-control study of PD in the Central Valley of California, showed that *FokI* polymorphism was linked to cognitive decline in PD [79].

In 2015, a study in California with higher ultraviolet radiation levels than in previous studies of VDR gene polymorphisms showed that the major allele *TaqI* TT genotype and the *ApaI* GG genotype are associated with decreased risk of PD [80]. However, some studies did not find any association between the VDR genotypes (*BsmI*, *FokI*, *ApaI*, and *TaqI* loci) and PD risk [61, 81]. The different results may be explained by a number of reasons. First, the effect of VDR gene polymorphism on PD risk may be related to the vitamin D levels. Second, these studies involved different ethnicities, environmental factors, gene-gene and gene-environment interactions, or small sample sizes. Therefore, future studies should shift to the interactions of vitamin D levels and VDR gene polymorphisms in PD, and take into account the environmental factors.

### The relationship between vitamin D level and clinical manifestations of PD patients

There is accumulating evidence that the PD patients have an increased prevalence of osteoporosis and osteopenia [82–84], and PD is recognized as a cause of secondary osteoporosis [85]. In a study conducted in Korea, researchers found that 6542 (18.3%) of 35,663 PD patients experienced osteoporosis, and that fractures occurred most commonly within 6 months after PD onset and decreased after 3 years from PD diagnosis [86]. Other studies have shown that bone loss and fractures in PD patients are multifactorial [87–89], with causes including vitamin D deficiency [90]. As PD patients experience more bone loss, more falls and more fractures (particularly at the hip) [91] than controls, osteoporosis should be screened and treated early [51, 82, 84, 92–94], particularly for older female patients within 3 years of PD diagnosis [86]. A meta-analysis of randomized controlled trials found that daily supplementation of 700 IU to 1000 IU of vitamin D could reduce the risk of falls by 19%, and that this advantage may not be dependent on additional calcium supplementation [95].

Many recent studies have confirmed the significant negative correlation between the severity of PD evaluated using the Hoehn & Yahr scale or Unified Parkinson’s Disease Rating Scale (UPDRS) and the circulating serum 25(OH)D levels [53–57, 96]. Prospective observational studies have also found a negative association between the vitamin D status at baseline and severity of PD motor symptoms during disease progression [41, 97]. Therefore, supplementation of vitamin D may delay the worsening of symptoms in PD patients. A cross-sectional, observational study supported the relationship between postural balance and serum vitamin D levels. Further analysis showed that among balance measures, vitamin D levels were associated with an automatic posture response to backwards translation, particularly with response strength and weight-bearing asymmetry [98].

PD patients often ignore nonmotor symptoms, which may, however, have been present for years before diagnosis. A large population-based sample of French older people found a strong relationship of lower 25(OH)D concentrations with cognitive decline, as well as increased risk of dementia and AD over 12 years of follow-up [99]. Likewise, in a sample of PD patients without cognitive impairment, higher vitamin D levels were associated with better cognition and mood [100]. The impact of vitamin D on cognition can partially be explained by its effect on amyloid beta (Aβ) [101], which has been shown to deposit in PD as well, probably leading or contributing to cognitive decline [102]. Interestingly, vitamin D has been reported to affect the Aβ-producing enzymes BACE1 and γ-secretase to reduce Aβ anabolism and elevate Aβ catabolism. Furthermore, vitamin D3 could reduce the cytotoxicity of Aβ peptide by ameliorating the decrease of the sphingosine-1-phosphate/ceramide ratio caused by Aβ [103]. A randomized double-blind trial found that vitamin D supplementation can effectively reduce the levels of Aβ, amyloid precursor protein (APP), BACE1, APP mRNA, and BACE1 mRNA [104]. Vitamin D and its receptors are important components of neuronal amyloid processing pathways [105]. Mayne et al. found that vitamin D deficiency may affect synaptic plasticity, leading to a decline of cognition [31]. Studies have shown that vitamin D signaling can affect the expression of *L*-type voltage-gated calcium channels, which are involved in neurotransmitter release, neuronal excitability change, learning and memory, etc. [31, 106]. Treatment of aging rodents with high-dose vitamin D3 could prevent cognitive decline and enhance hippocampal synaptic excitability [107].

Many studies have shown that brain regions involved in regulating olfactory function are closely related to cognitive decline, and the severity of olfactory disorder in PD patients may precede dementia [108–110]. In 2018, Kim et al. firstly demonstrated that the 25(OH)D_3_ levels were correlated with the severity of olfactory dysfunction in PD [111]. According to the Braak model of neuropathological staging of PD, the early stages 1 and 2 start from the medulla and the olfactory bulb [112], supporting a relationship between vitamin D and early PD. In addition to being associated with dementia and olfactory function in PD patients, serum 25(OH)D_3_ concentrations can also affect the gastric emptying time [113] and orthostatic hypotension [114].

### Preventive and therapeutic effects of vitamin D in PD

The pathophysiology of PD is affected by 1,25(OH)_2_D_3_ via genomic (Table 1) and non-genomic routes (rapid vitamin D-dependent membrane-associated effects) [120]. 1,25(OH)_2_D_3_ can increase or decrease the expression of a number of genes, thereby affecting intracellular signaling pathways. Recent pieces of evidence suggest that there is an inverse correlation between vitamin D concentrations and PD risk.

**Table 1:** Effects of 1,25-dihydroxyvitamin D_3_ exposure on gene expression in PD

Gene name	Gene location	Involvement in brain function	Expression change
*C-Ret* [115]	10q11.2	Neuroprotective effects and antioxidation	Up
*GDNF* [115]	5p13	Neuroprotective effects and antioxidation; dopaminergic neurotransmission	Up
*Nurr1* [116, 117]	2q22–23	DA neuronal differentiation and maturation	Up
*p57kip2* [116, 117]	11p15.5	DA neuronal differentiation and maturation	Up
*SLC30A10* [118]	2q32.3	Maintenance of homeostasis of calcium, zinc, iron and manganese	Up
*SLC39A2* [118]	14q11.2	Maintenance of homeostasis of calcium, zinc, iron and manganese	Down
*TH* [119]	11p15	Dopaminergic neurotransmission	Up

*C-Ret* proto-oncogene tyrosine-protein kinase receptor Ret, *GDNF* glial cell-derived neurotrophic factor, *TH* tyrosine hydroxylase

### 1,25(OH)_2_D_3_ affects PD by genomic actions mediated by VDR

#### Neuroprotective effects of vitamin D

**Fig. 2:**
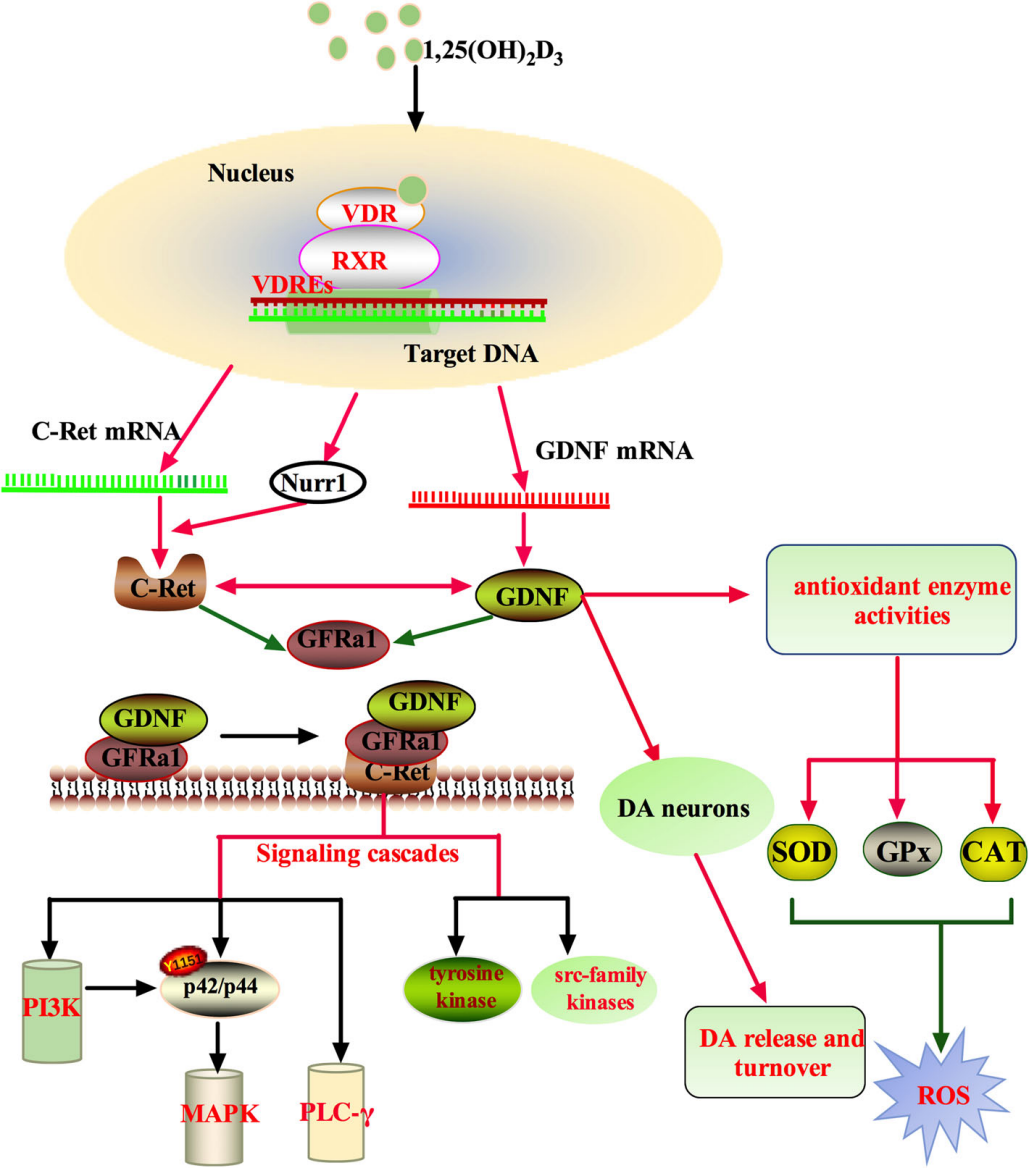
1,25(OH)_2_D_3_ exerts neuroprotective effects via GDNF. CAT, catalase; C-Ret, proto-oncogene tyrosine-protein kinase receptor Ret; DA, dopaminergic; GFR α1, GDNF family receptor alpha 1; GPx, glutathione peroxidase; MAPK pathway, p42/p44 mitogen-activated protein kinase pathway; PLC-γ pathway, phospholipase Cγ pathway; PI3K pathway, phosphoinositide 3-kinase pathway; ROS, reactive oxygen species; RXR, retinoid X receptor; SOD, superoxide dismutase; VDR, vitamin D receptor; VDREs, vitamin D response elements. The arrows indicate signaling components that are either enhanced (red arrows) or reduced (green arrows)

Glial cell-derived neurotrophic factors (GDNFs) facilitate neuronal regrowth and protect dopaminergic nerve terminals, which make them a very promising candidate for neuro-restoration therapy of PD [121, 122]. GDNF binds to the GDNF family receptor alpha 1 (GFRa1) and then associates with the proto-oncogene tyrosine-protein kinase receptor Ret (C-Ret). This complex enables GDNF to exert intracellular signaling in DA neurons [115]. However, GDNF cannot pass the blood-brain barrier, and injecting GDNF into the CNS has many negative effects [121]. As a fat-soluble vitamin, 1,25(OH)_2_D_3_ can pass the blood-brain barrier, which consolidates the importance of this hormone in PD [39]. Upon VDR binding, 1,25(OH)_2_D_3_ directly upregulates the transcription of genes targeted by C-Ret and GDNF. There is a positive feedback between GDNF and C-Ret, and both can suppress GFRa1 production [115]. Depletion of 1,25(OH)_2_D_3_ results in decreased expression of GDNF, Nurr1 and p57kip2 [123, 124], which may alter the differentiation and maturation of DA neurons in the developing rat brain [116, 117]. Nurr1 is also crucial for the expression of C-Ret [115], which in turn triggers Src-family kinases and tyrosine kinase, activating several downstream signaling cascades, including the phosphoinositide 3-kinase (PI3K) pathway, the phospholipase Cγ (PLC-γ) pathway, and the p42/p44 mitogen-activated protein kinase (MAPK) pathway [125, 126]. The activation of the MAPK pathway requires a basal activity of the PI3K pathway [126]. The activation of these pathways may promote the survival and differentiation of midbrain DA neurons (Fig. 2). In conclusion, 1,25(OH)_2_D_3_ exerts its neuroprotective effects by increasing the expression of GDNF gene and then activating several downstream intracellular signaling cascades.

**Fig. 3:**
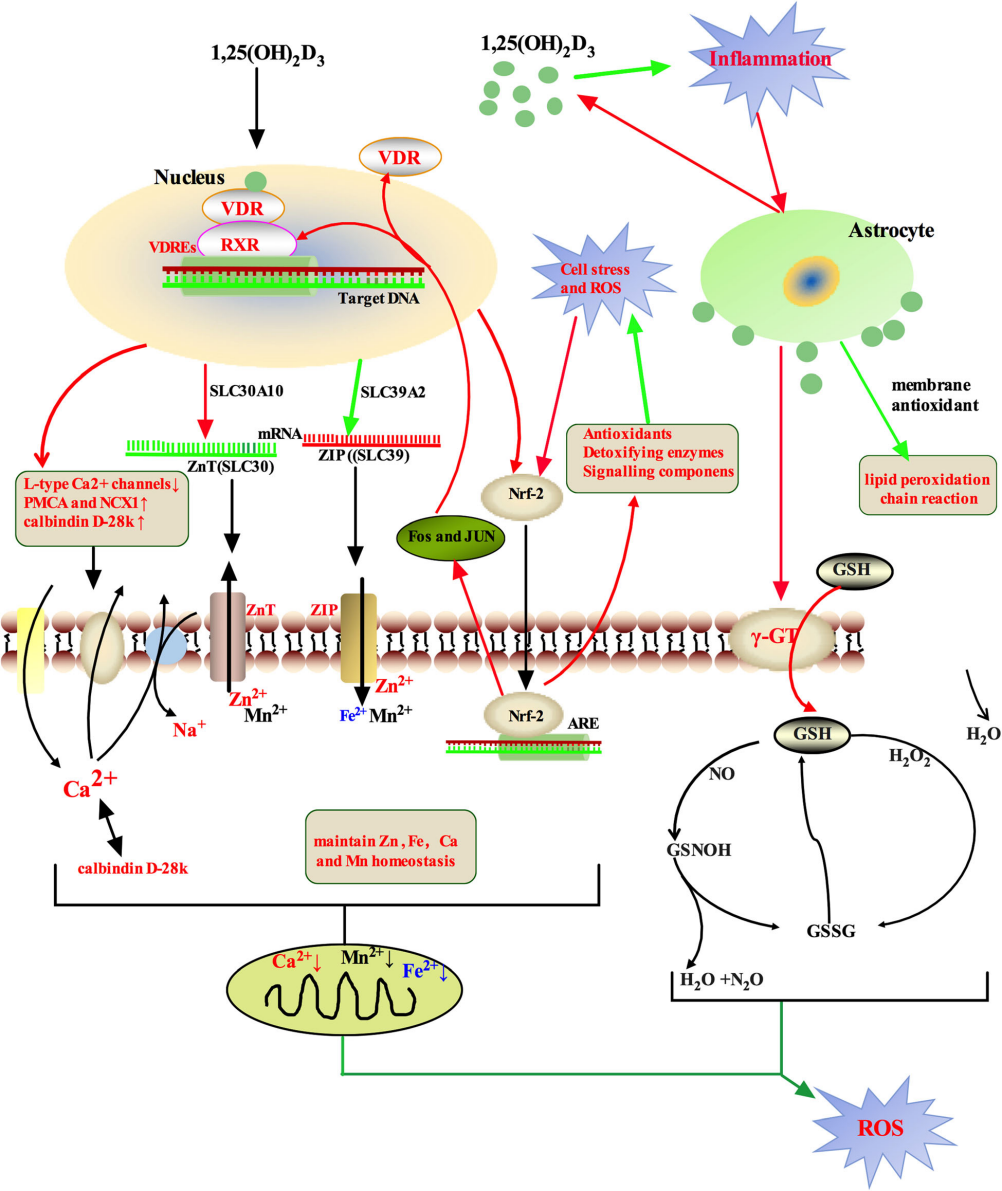
1,25(OH)_2_D_3_ also exerts neuroprotective effects through genomic and/or non-genomic activation. ARE, antioxidant response element; GFR α1, GDNF family receptor alpha 1; γ-GT, gamma-glutamyl transferase; GSH, glutathione; GSNOH, S-nitrosoglutathione; RXR, retinoid X receptor; ROS, reactive oxygen species; PMCA, plasma membrane Ca^2+^ ATP-ase; Nrf2, nuclear factor erythroid 2-related factor 2; VDR, vitamin D receptor; VDREs, vitamin D response elements. The arrows indicate signaling components that are either enhanced (red arrows) or reduced (green arrows)

In addition, 1,25(OH)_2_D_3_ is also an antioxidant, which may further contribute to its neuroprotective effects. Studies have demonstrated that 1,25(OH)_2_D_3_ increases the expression of GDNF, a powerful antioxidant that can reduce reactive oxygen species (ROS). GDNF markedly increases the levels of superoxide dismutase, glutathione peroxidase and catalase in the striatum (Fig. 2) [127]. In addition to the upregulation of GDNF expression, 1,25(OH)_2_D_3_ can also exert its antioxidant effect through genomic and/or nongenomic activation. Under inflammatory stimulation, microglial cells can produce 1,25(OH)_2_D_3_ *in situ*, where it potentiates the mRNA expression of gamma-glutamyl transferase (γ-GT) and γ-GT activity induced by proinflammatory stimuli. γ-GT mediates the import of glutathione (GSH) into the cell, after which the intracellular GSH reduces the production of reactive nitrogen species and hydrogen peroxide [128]. In addition, 1,25(OH)_2_D_3_ also increases the expression of the nuclear factor erythroid 2-related factor 2 (Nrf2). When ROS rise, they bind to antioxidant response elements (AREs) in the nucleus, enhancing the expression of antioxidant genes, detoxifying enzymes and various signaling components. By increasing the expression of Fos and JUN, Nrf2 also increases the expression of both VDR and RXR [129]. Moreover, 1,25(OH)_2_D_3_ can directly inhibit lipid peroxidation as a membrane antioxidant, which protects the membranes of normal cells from ROS-induced oxidative damage [130, 131]. Therefore, 1,25(OH)_2_D_3_ contributes to the enhancement of antioxidative systems by increasing the expression of GDNF, γ-GT and Nrf2 (Fig. 3).

It has also been reported that 1,25(OH)_2_D_3_ has anti-inflammatory properties. It can attenuate pro-inflammatory and upregulate anti-inflammatory processes [132]. In the 6-OHDA-induced PD model, pre- or post-treatment with 1,25(OH)_2_D_3_ reduced tissue immunopositivity for TNF-α, partially restored tyrosine hydroxylase (TH) immunoreactivity, and prevented the decrease of VDR immunoreactivity in the lesioned striatum [133, 134]. Cell culture studies revealed that the increased intracellular free calcium can induce the aggregation of α-synuclein, and proved that the increase of intracellular calcium and oxidative stress can act cooperatively to promote α-synuclein aggregation [135–138]. By reducing the expression of *L*-type Ca^2+^ channels and increasing the expression of the plasma membrane Ca^2+^ ATP-ase, NCX1, anti-apoptotic factor Bcl-2 and buffering protein calbindin D28k, 1,25(OH)_2_D_3_ can maintain the low cytosolic Ca^2+^ concentrations and thereby protect against Ca^2+^-induced oxidative damage in SN dopaminergic neurons [106, 129]. High concentrations of divalent metal ions exhibit toxic effects that may cause an elevation of ROS levels and mitochondrial dysfunction, and even induce neuronal cell death. Notably, 1,25(OH)_2_D_3_ can maintain zinc, iron and manganese homeostasis by regulating the expression of related genes. It can transactivate *SLC30A10* to increase the expression of zinc and manganese transporter ZnT10. It can also decrease the expression of *SLC39A2*, which encodes the ZIP (SLC39) protein implicated in zinc, iron and/or manganese transport. The ZnT (SLC30) protein reduces the cytoplasmic concentrations of metal ions, while ZIP (SLC39) transporters lead to an increase (Fig. 3) [118]. In short, 1,25(OH)_2_D_3_ can maintain the homeostasis of calcium, zinc, iron and manganese by regulating the expression of some genes, thereby reducing oxidative stress and mitochondrial damage.

### Vitamin D is closely associated with dopaminergic neurotransmission

Studies have shown that 1,25(OH)_2_D_3_ and VDR are directly involved in regulating the expression of genes in dopaminergic neurons [139]. Many studies have found that VDR protein levels and TH expression are enhanced in the brains of rats following 1,25(OH)_2_D administration [119, 140]. Notably, TH is the rate-limiting enzyme of dopamine synthesis. It has been reported that a likely mediator of the regulation of TH expression by vitamin D is N-cadherin [141]. In line with these findings, researchers found that pre- or post-treatments with 1,25(OH)_2_D_3_ restored the decreased DA content, and increased the expression of TH and dopamine transporter in 6-OHDA-lesioned rats according to striatal neurochemical and immunohistochemical assays [133]. GDNF can act directly on DA neurons to enhance their activity and increase DA release [127]. In a word, 1,25(OH)_2_D_3_ may participate in dopaminergic neurotransmission via TH expression regulation and the direct effect of GDNF on DA neurons, which mediates the relationship between vitamin D concentrations and the severity of PD.

### 1,25(OH)_2_D_3_ affects PD by rapid vitamin D-dependent membrane-associated effects

Protein disulfide isomerase 3 (PDIA3), also known as the endoplasmic reticulum stress protein 57 (ERp57), acts as another 1,25(OH)_2_D_3_ membrane receptor [142]. Compared to the kidney and liver, PDIA3 is highly expressed in all types of brain cells and can be considered as the main VDR in the brain [143]. It is a multifunctional protein that can not only control the quality of protein processing, but also maintain Ca^2+^ homeostasis and regulate cellular stress responses [144, 145]. In the PD model induced by 6-OHDA, the level of PDIA3 protein in the striatum is increased, which may be a cellular response to oxidative stress. In this case, PDIA3 may act as a chaperone to prevent the misfolding and aggregation of α-synuclein [144]. After generation of ROS by 6-OHDA, protein oxidation occurs first, and early in the protein oxidation process, the PDIA3 rapidly forms juxtanuclear aggresome-like structures (ERp57/DNA) in dopaminergic cells, which may induce downstream sequelae such as the unfolded protein response, cell stress, and apoptosis [146, 147]. ERp57 has an affinity for Ref-1, which has a synergistic effect and jointly regulates the gene expression mediated by redox-sensitive transcription factors and the adaptive responses of cells to oxidative damage [147]. As a result, it is likely that vitamin D functions in the PD through PDIA3.

### Vitamin D supplementation in PD patients

The most important studies on vitamin D supplementation in PD patients are shown in Table 2. An interventional trial supported the role of vitamin D in postural balance of PD patients and suggested that daily supplementation of vitamin D could improve the balance of younger PD patients [148]. Other studies have also confirmed that supplemental 25(OH)D has beneficial effects on strength and balance in older adults [149]. Therefore, there is a debate on whether vitamin D supplementation can specifically delay the progression of motor symptoms in PD patients, or only lead to a non-specific improvement in muscle strength and balance. However, the complex automatic postural response not only requires muscle function, but also involves the spinal cord, midbrain/brainstem, and cerebellum/basal ganglia/cerebral cortex [150, 151]. In addition, vitamin D_3_ supplementation has an age-dependent effect on PD [148]. Another randomized controlled trial of vitamin D supplementation found that vitamin D_3_ supplementation may retard the progression of PD for a short period in patients with *FokI* CT and TT genotypes [41]. Therefore, the extensive roles of vitamin D in the skeletal muscle and neural systems suggest that vitamin D can affect the symptoms of PD.

**Table 2:** Vitamin D_3_ supplementation in PD patients

Author	Country	Type of study	Number of participants (T/C)	Intervention	Follow-up	Adverse events
Hiller et al. 2018 [148]	USA	RCT	27/24	Vitamin D_3_ 10,000 IU/day	16 weeks	None
Suzuki et al. 2013 [41]	Japan	RCT	56/58	Vitamin D_3_ 1200 IU/day	12 months	None

*T* treatment group, *C* control group, *PD* Parkinson’s disease

## Conclusions and future directions

In summary, the most consistent view at present is that the concentration of vitamin D is low in PD patients. Higher vitamin D concentrations are linked to reduced risk and severity of PD, as well as better cognition and mood of the patients. Furthermore, the VDR gene phenotypes may influence the risk and severity of PD, as well as the effect of vitamin D supplementation in PD patients. Although there are limited data on the effectiveness of vitamin D_3_ supplementation in PD patients, related studies have highlighted the effectiveness of vitamin D_3_ supplementation in preventing osteoporotic fractures in the aging population and retarding the progression of PD for a short period.

A recent study has found that vitamin D has the potential to be used as a biomarker for PD [152], inspiring great interest in the relationship between PD and vitamin D. Vitamin D can improve protein homeostasis and slow down the aging process [153], but vitamin D insufficiency is prevalent worldwide [48]. Moreover, vitamin D supplementation is readily available, affordable and safe. The earliest detectable side-effects of vitamin D supplementation are hypercalciuria and hypercalcemia, which are only a concern when 25(OH)D levels exceed 88 ng/mL (220 nmol/L) [154, 155]. Vitamin D supplementation in PD patients at a dose of 1200 IU/day for 12 months [41] or 10,000 IU/day for 16 weeks [148] did not lead to obvious adverse events such as hypercalcemia (Table 2). Therefore, vitamin D supplementation in PD patients seems to be promising, although the dose of vitamin D that may cause toxicity remains unclear. Despite the limited long-term safety data, in 2010, the Institute of Medicine (IOM) defined a safe upper limit dosage for vitamin D of 4000 IU/day, although practitioners should keep in mind the intake of other dietary supplements [156]. The possibility of neuroprotection is the most exciting aspect of vitamin D therapy in PD. Considering the neuroprotective effects of vitamin D and the role of vitamin D in dopaminergic neurotransmission, interventional prospective studies on vitamin D supplementation in PD patients should be conducted in the future.

## Data Availability

All data generated or analyzed during this study are included in this published article.

## Abbreviations

ARE: Antioxidant response element
Aβ: Amyloid beta
APP: Amyloid precursor protein
C-Ret: Proto-oncogene tyrosine-protein kinase receptor Ret
DA: Dopaminergic
ERp57: Endoplasmic reticulum stress protein 57
γ-GT: Gamma-glutamyl transferase
GSH: Glutathione
GDNF: Glia-derived neurotrophic factors
GFRa1: GDNF family receptor alpha 1
MAPK pathway: p42/p44 mitogen-activated protein kinase pathway
Nrf2: Nuclear factor erythroid 2-related factor 2
PD: Parkinson’s disease
PLC-γ pathway: Phospholipase Cγ pathway
PMCA: Plasma membrane Ca^2+^ ATP-ase
PI3K pathway: Phosphoinositide 3-kinase pathway
PDIA3: Protein disulfide isomerase 3
ROS: Reactive oxygen species
RXR: Retinoid X receptor
SN: Substantia nigra
TH: Tyrosine hydroxylase
UPDRS: Unified Parkinson’s Disease Rating Stage
VDR: Vitamin D receptor
VDREs: Vitamin D response elements
25(OH)D: 25-hydroxyvitamin D
1,25(OH)_2_D_3_: 1,25-dihydroxyvitamin D_3_
1-OHase: 25-hydroxyvitamin D-1α-hydroxylase
24-OHase: 25-hydroxyvitamin D-24-hydroxylase
